# Interim 2024/25 influenza vaccine effectiveness: eight European studies, September 2024 to January 2025

**DOI:** 10.2807/1560-7917.ES.2025.30.7.2500102

**Published:** 2025-02-20

**Authors:** Angela MC Rose, Héloïse Lucaccioni, Kimberly Marsh, Freja Kirsebom, Heather Whitaker, Hanne-Dorthe Emborg, Amanda Bolt Botnen, Mark G O’Doherty, Francisco Pozo, Safraj Shahul Hameed, Nick Andrews, Mark Hamilton, Ramona Trebbien, Karina Lauenborg Møller, Diogo FP Marques, Siobhan Murphy, Ross McQueenie, Jamie Lopez-Bernal, Simon Cottrell, Magda Bucholc, Esther Kissling, European IVE group, Members of the European IVE group, Anna Parys, Arne Witdouck, Benedicte Delaere, Benédicte Lissoir, Catherine Quoidbach, Catherine Sion, Claire Brugerolles, Deborah De Geyter, Dylan Lievens, Eva Bernaert, Eveline Van Honacker, Evelyn Petit, Francesco Genderini, François Dufrasne, Isabel Leroux-Roels, Katelijne Flore, Katty Renard, Koen Magerman, Laurane De Mot, Lucie Seyler, Marc Bourgeois, Marieke Bleyen, Marie-Pierre Parsy, Marijke Reynders, Marlies Blommen, Mathil Vandromme, Melanie Delvallee, Natasja Detillieu, Nathalie Bossuyt, Nicolas Dauby, Pascal De Waegemaeker, Pierre Struyven, Reinout Naesens, Sandra Koenig, Sarah Denayer, Sebastien Fierens, Siel Daelemans, Sigi Van Den Wijngaert, Silke Ternest, Stephanie Buylla, Sven Hanotaux, Veerle Penders, Xavier Holemans, Yinthe Dockx, Yves Lafort, Zvjezdana Lovrić Makarić, Goranka Petrović, Sanja Kurečić Filipović, Bernard Kaić, Vesna Višekruna Vučina, Ivan Mlinarić, Irena Tabain, Svjetlana Karabuva, Petra Tomaš Petrić, Rok Čivljak, Ivan Krešimir Lizatović, Borna Grgić, Elizabeta Dvorski, Nives Bubnjar, Mia Breški, Iva Pem Novosel, Jens Nielsen, Noémie Sève, Caroline Guerrisi, Thierry Blanchon, Titouan Launay, Alessandra Falchi, Shirley Masse, Leïla Renard, Marie Chazelle, Sylvie van der Werf, Vincent Enouf, Bruno Lina, Martine Valette, Anthony Nardone, Marlena Kaczmarek, Nathalie Nicolay, Sabrina Bacci, Ralf Duerrwald, Annika Erdwiens, Carolin Hackmann, Kristin Tolksdorf, Silke Buda, Ute Preuss, Marianne Wedde, Janine Reiche, Barbara Biere, Beatrix Oroszi, Gergő Túri, Viktória Velkey, Katalin Krisztalovics, Katalin Kristóf, Bánk Gábor Fenyves, Csaba Varga, Márta Knausz, Bernadett Burkali, István Zsolt, Melinda Kiss-Fekete, Zoltán Péterfi, Lisa Domegan, Róisín Duffy, Margaret Fitzgerald, Adele McKenna, Charlene Bennett, Ligita Jančorienė, Fausta Majauskaitė, Auksė Mickienė, Monika Kuliešė, Tanya Melillo, John-Paul Cauchi, Adam Meijer, Dirk Eggink, Mariëtte Hooiveld, Eline In ‘t Velt, Marit de Lange, Rianne van Gageldonk-Lafeber, Ana Paula Rodrigues, Ausenda Machado, João Santos, Verónica Gomez, Camila Henriques, Licínia Gomes, Miguel Lança, Daniela Dias, Nuno Verdasca, Raquel Guiomar, Mihaela Lazar, Odette Popovici, Esteban Pérez Morilla, Virtudes Gallardo García, Miriam García Vázquez, Inés Guiu Cañete, Mª Olga Hidalgo Pardo, María Torres Juan, Eva Rivas Wagner, Nieves López González-Coviella, M Ángeles Rafael de la Cruz López, Carmen Román Ortiz, Jacobo Mendioroz, Luca Basile, Ana Sofía Lameiras Azevedo, Paloma Botella Rocamora, María Cecilia Puerto Hernández, Marina Paula Martins Agostinho Simoes Fernandes, Olaia Pérez-Martínez, María-Dolores Chirlaque, Blanca Andreu Ivorra, Carmen Quiñones Rubio, Violeta Ramos, Ninoska Lopez, Daniel Castrillejo, Francisco Javier de la Vega-Olías, Marcos Lozano, Gloria Pérez-Gimeno, Susana Monge, Iván Martínez-Baz, Camino Trobajo Sanmartin, Jesús Castilla, Aitziber Echeverría, Nerea Egüés, Guillermo Ezpeleta, Ana Navascués, Leticia Armendáriz, Carmen Ezpeleta, Neus Latorre-Margalef, Dorina Ujvari, Julia Stowe, Alec Cobbold, Katja Hoschler, Beatrix Kele, Maria Zambon, Jim McMenamin, Chris Robertson, Jana Zitha, Catherine Moore, Panoraia Kalapotharakou, Tim Jones, Anastasia Couzens, Simon DeLusignan, Rosalind Goudie, Gavin Jamie, Elizabeth Button

**Affiliations:** 1 Epiconcept, Paris, France; 2 These authors contributed equally as first authors; 3 These authors contributed equally to the writing of the paper; 4 Public Health Scotland, NHS Scotland, Glasgow, United Kingdom; 5 UK Health Security Agency, London, England; 6 Department of Infectious Disease Epidemiology and Prevention, Statens Serum Institut, Copenhagen, Denmark; 7 Department of Virology and Microbiological Preparedness, Statens Serum Institut, Copenhagen, Denmark; 8 Public Health Agency, Belfast, United Kingdom; 9 National Centre for Microbiology, Institute of Health Carlos III, Madrid, Spain; 10 Consortium for Biomedical Research in Epidemiology and Public Health (CIBERESP), Madrid, Spain; 11 Data Integration and Analyses, Statens Serum Institut, Copenhagen, Denmark; 12 Public Health Wales, Cardiff, Wales; 13 European Influenza Vaccine Effectiveness (IVE) group members are listed under Collaborators

**Keywords:** influenza, vaccine effectiveness, multicentre study, test-negative design, Europe

## Abstract

The 2024/25 influenza season in Europe is currently characterised by co-circulation of influenza A(H1N1)pdm09, A(H3N2) and B/Victoria viruses, with influenza A(H1N1)pdm09 predominating. Interim vaccine effectiveness (VE) estimates from eight European studies (17 countries) indicate an all-age influenza A VE of 32–53% in primary care and 33–56% in hospital settings, with some signals of lower VE by subtype and higher VE against influenza B (≥ 58% across settings). Where feasible, influenza vaccination should be encouraged and other prevention measures strengthened.

For the northern hemisphere, the World Health Organization (WHO) recommended the following 2024/25 influenza virus strains for egg-based vaccines: an A/Victoria/4897/2022 (H1N1)pdm09-like, an A/Thailand/8/2022 (H3N2)-like and a B/Austria/1359417/2021 (B/Victoria lineage)-like virus for trivalent vaccines. For cell culture- or recombinant-based vaccines, the WHO recommended inclusion of an A/Wisconsin/67/2022 (H1N1)pdm09-like and an A/Massachusetts/18/2022 (H3N2)-like virus, with the same influenza B virus component as for egg-based vaccines. For both egg- and non-egg-based vaccines, the WHO recommended inclusion of an additional B/Phuket/3073/2013 (B/Yamagata lineage)-like virus for quadrivalent vaccines [1].

## European primary care and hospital-based studies to measure influenza vaccine effectiveness in 2024/25

We report interim results from five single- and three multi-country studies (17 countries), including both primary care and hospital settings, to help guide influenza prevention and control measures for the rest of the 2024/25 season and to inform preparation for the 2025/26 season.

**Figure 1:**
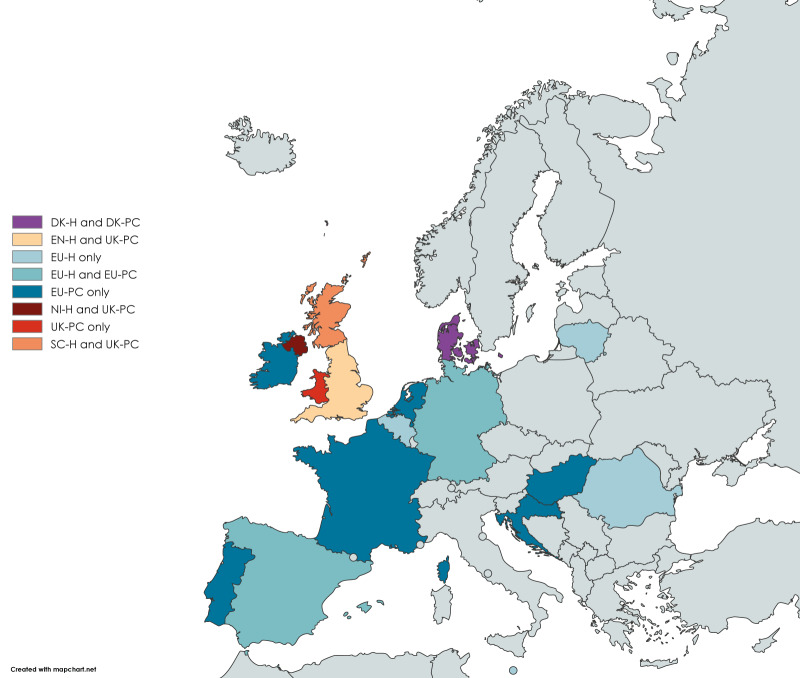
European countries contributing results^a^ for interim influenza vaccine effectiveness, influenza season 2024/25 (n = 17)

The primary care (PC) studies were conducted in Denmark (DK-PC), the United Kingdom (UK) (UK-PC: four countries), and through the European Union (EU) Vaccine Effectiveness, Burden and Impact Studies (VEBIS) multi-country primary care network (EU-PC: eight of 10 countries contributing to the interim analysis). The hospital setting (H) studies were conducted in Denmark (DK-H), England (EN-H), Northern Ireland (NI-H), Scotland (SC-H), and through the EU VEBIS multi-country hospital network (EU-H: six of 10 countries contributing to the interim analysis) (Figure 1).

DK-H/DK-PC: Denmark hospital and primary care studies; EN-H: England hospital study; EU-H/EU-PC: European Union hospital-/primary care-based multi-country VEBIS studies; NI-H: Northern Ireland hospital study; SC-H: Scotland hospital study; UK-PC: United Kingdom primary care multi-country study; VEBIS: Vaccine Effectiveness, Burden and Impact Studies.

^a^ Countries contributing to EU-H but not included in the analysis (as too few cases remained after applying exclusions and restrictions): Croatia, Hungary, Ireland, Portugal. Countries contributing to EU-PC but not included in the analysis: Romania, Sweden.

## Study design and vaccine effectiveness analyses

The EU VEBIS project has been estimating influenza vaccine effectiveness (VE) through multicentre primary care and hospital studies since the 2022/23 influenza season. Before this, many VEBIS study sites (primary care and hospital) participated in the Influenza – Monitoring Vaccine Effectiveness in Europe (I-MOVE) network, measuring annual influenza VE from 2008/09 to 2021/22 [2,3]. The UK and Denmark were I-MOVE partners until 2021/22 and have estimated influenza VE in single-country studies since 2006 and 2009, respectively. We have jointly published interim season influenza VE results since 2017/18 [4]. All studies use the test-negative design [5], with methods previously described [6–9]. There are some differences in recruitment practice or collection of data by study; we summarise methods by study in Table 1.

**Table 1:** Summary of methods for the eight European interim influenza vaccine effectiveness studies, influenza season 2024/25 (n = 17 countries)

Study characteristics	Study							
	DK-PC	EU-PC	UK-PC	DK-H	EN-H	EU-H	NI-H	SC-H
Period	30 Sep 2024– 31 Jan 2025	4 Oct 2024– 14 Jan 2025	30 Sep 2024– 10 Jan 2025	30 Sep 2024– 31 Jan 2025	30 Sep 2024– 5 Jan 2025	15 Oct 2024– 17 Jan 2025	29 Sep 2024– 20 Jan 2025	1 Oct 2024– 21 Jan 2025
Setting	Non-hospitalised patients^a^	Primary care	Primary care	Hospital	Hospital	Hospital	Hospital	Hospital
Location	DK	HR, FR, DE, HU, IE, NL, PT, ES	EN, NI, SC, WA	DK	EN	74 hospitals in BE, DE, ES, LT, MT, RO	NI	SC
Design	TND	TND	TND	TND	TND	TND	TND	TND
Data source(s)	Data linkage of Danish Microbiology Database, the Danish Vaccination Register and the Danish National Discharge Register	Sentinel physicians and laboratories; in some sites data linkage to electronic health records	Sentinel physicians and laboratories; in some sites data linkage to vaccine registries	Data linkage of Danish Microbiology Database, the Danish Vaccination Register and the Danish National Discharge Register	Data linkage of laboratory surveillance, the Immunisations Information System, and the Secondary Uses Service	Hospital charts, vaccine registers, interviews with patients, laboratory records	Linkage of vaccination status from the Northern Ireland Vaccine Management System, influenza tests from the regional laboratory surveillance system, and administrative admissions data from Health and Social Care information systems	National patient-level dataset based on GP records, Electronic Communication of Surveillance in Scotland ECOSS (all virology testing national database), Rapid Preliminary Inpatient Data RAPID (Scottish hospital admissions data), National Records of Scotland NRS (death certification), National Clinical Data Store NCDS (vaccination events in Scotland)
Age groups of study population	All ages	≥ 6 months	≥ 2 years	All ages	≥ 2 years	All ages	Adults ≥ 18 years	≥ 2 years
Case definition for patient recruitment	Sudden onset of symptoms with fever, myalgia and respiratory symptoms^b^	EU ARI^c^ or EU ILI^c^	ARI	Sudden onset of symptoms with fever, myalgia and respiratory symptoms among hospitalised patients	ARI-coded hospital visit with a swab taken 14 days before to 2 days after admission	SARI (hospitalised person with fever cough, or shortness of breath) at admission or within 48 h after admission); some countries recruit those with fever or cough; some include those only with fever and cough	Patients with a positive influenza test performed either up to 7 days before admission or up to 1 day after admission. Limited to emergency care^d^	Patients with a positive influenza test 14 days before admission or within 48 h of admission. Limited to emergency care^d^
Selection of patients	At practitioner's/ clinician's judgement	Systematic	At practitioner's/ clinician's judgement	At practitioner's/ clinician's judgement	Exhaustive	Exhaustive (DE, LT, MT, RO, some hospitals in ES). Systematic (BE, ES; some hospitals in BE: exhaustive on either 1 or 2 days per week, depending on workload)	Exhaustive (all patients who fit the case definition and are captured via the linkage of the named datasets)	Exhaustive (all patients who fit the case definition above and are captured via the linkage of the named datasets)
Vaccine types used nationally or in the study^e,f^	In the population: 50% QIV, 45% aQIV (offered to individuals ≥ 70 years), 5% QIV-HD (offered to individuals ≥ 65 years)	In the study among controls: 48% QIV, 13% aQIV, 10% QIV-HD, 8% QIVc, 8% LAIV (trivalent and quadrivalent), 2% TIV, and 12% unknown	In the study among controls: ages 2–17 years 90% LAIV, 5% QIVc, 5% unknown; ages 18–64 years 67% QIVc, 2% aQIV, 1% QIV, 0.2% QIV-HD, 29% unknown; ages ≥ 65 years 72% aQIV, 2% QIV-HD, 1% QIVc, 0.1% QIV, 25% unknown	In the population: 50% QIV, 45% aQIV (offered to individuals ≥ 70 years), 5% QIV-HD (offered to individuals ≥ 65 years)	In the study among controls: ages 2–17 years 80% LAIV, 13% QIVc, 6% unknown; ages 18–64 years 84% QIVc, 6% aQIV, 4% QIV, 1% QIV-HD, 6% unknown; ages ≥ 65 years 88% aQIV, 4% QIV-HD, 3% QIVc, 5% unknown	In the study among controls: 45% QIV; 21% aQIV, 15% QIV-HD, 5% QIVc, 4% LAIV (trivalent and quadrivalent), 12% unknown	In the study among controls: 26.5% QIVc, 73.5% aQIV	23% QIVc; 77% aQIV
Variables of adjustment	Age group, sex, presence of chronic conditions, calendar time as month (Oct–Jan) or if possible, week	Age (modelled as RCS, age group or linear term depending on analysis), sex, presence of chronic conditions, onset date (RCS) and study site	Age group, sex, country, clinical risk status, calendar time as week (spline)	Age group, sex, presence of chronic conditions, calendar time as month (Oct–Jan) or if possible, week	Age group, region, clinical risk status, calendar time as week (spline)	Age (modelled as RCS, age group or linear term depending on analysis), sex, presence of chronic conditions, time (onset date as RCS or month of swab as categorical term) and study site	Age group, sex, month of the test, and Health and Social Care Trust	Age (spline), sex, number of clinical risk groups (0,1,2,3,4, ≥ 5), time (days, spline), setting (community or hospital) and deprivation quintile (SIMD)

aQIV: adjuvanted QIV; ARI: acute respiratory infection; BE: Belgium; DE: Germany; DK: Denmark; DK-H: DK hospital study; DK-PC: DK primary care study; EN: England; EN-H: EN hospital study; ES: Spain; EU: European Union; EU-H: EU hospital multicentre VEBIS study; EU-PC: EU primary care multicentre VEBIS study; FR: France; GP: general practitioner; HR: Croatia; HU: Hungary; IE: Ireland; ILI: influenza-like illness; LAIV: live attenuated influenza vaccine; LT: Lithuania; LRI: lower respiratory infection; MT: Malta; NI: Northern Ireland; NL: Netherlands; PT: Portugal; QIV: quadrivalent inactivated influenza vaccine; QIVc: cell-based QIV; QIVe: egg-grown QIV; QIV-HD: QIV – high-dose; RCS: restricted cubic spline; RO: Romania; SARI: severe acute respiratory infection; SARS-CoV-2: severe acute respiratory syndrome coronavirus 2; SC: Scotland; SC-H: SC hospital study; SIMD: Scottish Index of Multiple Deprivation; SE: Sweden; TIV: trivalent inactivated influenza vaccine; TND: test-negative design; UK: United Kingdom; UK-PC: UK primary care multicentre study; VE: vaccine effectiveness; VEBIS: VE, Burden and Impact Studies; WA: Wales.

^a^ Patients are seen by the GP, but also in emergency care.

^b^ This is the case definition for patient recruitment by sentinel GPs within DK-PC who follow the EU-ILI case definition (sudden onset of symptoms, AND at least one of: fever > 38° C, feverishness, malaise, headache, myalgia, AND at least one of: cough, sore throat, shortness of breath).

^c^ The EU-ARI definition is sudden onset of symptoms AND at least one of cough, sore throat, shortness of breath or coryza, AND a clinician’s judgement that the illness is due to an infection. The EU-ILI definition is sudden onset of symptoms AND at least one of: fever or feverishness, malaise, headache, myalgia AND at least one of: cough, sore throat, shortness of breath. Most EU-PC sites recruit according to the EU-ARI case definition, although there are some site-specific differences in ARI (and/or ILI) definitions for recruitment.

^d^ All patients entering hospital via an emergency department who were tested for influenza were assumed to have had an ARI symptom.

^e^ Vaccines were prepared from egg-grown vaccine viruses, non-adjuvanted and administered intramuscularly unless otherwise specified.

^f^ Where indicated, vaccine coverage among controls was used as representative of the source population from which the cases arose.

Briefly, three of the multicentre studies (EU-H, EU-PC and UK-PC) used prospective patient recruitment, while five studies used electronic database linkage (DK-H, DK-PC, EN-H, NI-H, SC-H). Patients presenting with influenza-like illness (ILI) or acute respiratory infection (ARI) symptoms in the primary care studies had nasopharyngeal or combined oro-nasopharyngeal specimens (or saliva specimens, in France) collected. In EU-H, patients admitted with severe ARI (SARI) symptoms were swabbed. In EN-H, NI-H and SC-H, all patients entering hospital via an emergency department who were tested for influenza were assumed to have had at least one ARI symptom. In each study, either all or a systematic selection of patients were swabbed, or the physician’s discretion was used to select patients for swabbing.

For influenza virus detection, samples were tested by reverse transcription (RT)-PCR for type A and type B viruses, followed by type A subtyping or B lineage determination. We defined cases as patients whose tests were positive for any influenza virus (sub)type; controls were those testing RT-PCR-negative for all influenza viruses. Most studies recruited all children and adults, while some applied age restrictions (Table 1).

We defined vaccinated patients as those having had the 2024/25 influenza vaccine at least 14 days before symptom onset. Those vaccinated < 14 days before symptom onset, or with unknown vaccination date, were excluded.

Most study countries (six from EU-PC, three from EU-H, and Denmark) selected all or a random sample of influenza virus-positive specimens for haemagglutinin genome segment and/or whole genome sequencing. Sequencing was followed by phylogenetic analysis to determine clade distribution, with results provided for both studies in Denmark together (DK-PC and DK-H).

## Statistical analysis

In each study, we calculated VE as 1 minus the adjusted ratio of the odds of vaccination in cases and controls, expressed as a percentage: VE = (1 − OR_a_) × 100. We used logistic regression to adjust for measured potential confounding variables (Table 1).

We estimated study-specific VE against any influenza, influenza A overall, and against influenza A(H1N1)pdm09, A(H3N2) and B. We performed sensitivity analyses using Firth’s method of penalised logistic regression (PLR) to assess small sample bias [10] for analyses with fewer than 10 cases or controls per parameter. We considered > 10% difference between the original and the PLR estimates as indicating small sample bias and do not show these estimates.

## Virus characteristics

**Figure 2:**
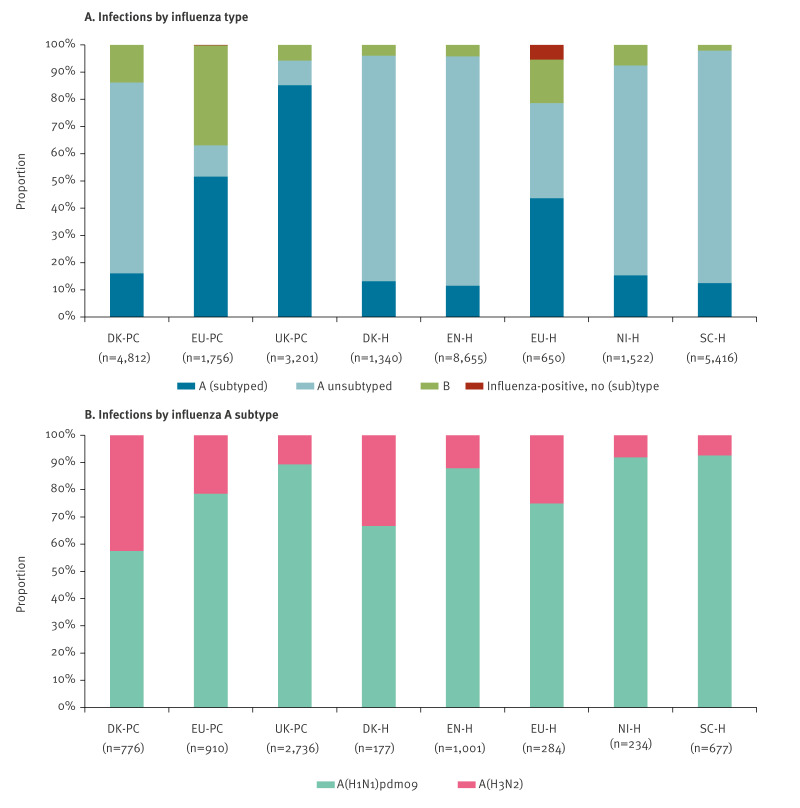
Proportion of influenza virus infections, eight European studies, interim influenza season 2024/25 (n = 27,170)^a^

DK-H: Denmark hospital study; DK-PC: Denmark primary care study; EN-H: England hospital study; EU: European Union; EU-H: EU hospital multicentre VEBIS study; EU-PC: EU primary care multicentre VEBIS study; PC: primary care; NI-H: Northern Ireland hospital study; SC-H: Scottish hospital study; UK-PC: United Kingdom multicentre primary care study; VEBIS: Vaccine Effectiveness, Burden and Impact Studies.

^a^ Includes 15 influenza A and B co-infections in DK-PC, 13 in EN-H, seven in EU-PC, six in UK-PC; six influenza A(H1N1)pdm09 and A(H3N2) co-infections in UK-PC, one in DK-PC and one in DK-H; and one influenza A(H1N1)pdm09 and B co-infection in DK-PC.

In this season, influenza A virus subtypes and influenza B co-circulated in Europe [11]. Influenza A(H1N1)pdm09 was the main subtype among all studies, ranging between 57% and 93% of influenza A subtypes (Figure 2). The proportion of influenza B cases varied considerably, from 2% in SC-H to 37% in EU-PC. Within the EU-PC and EU-H multicentre studies, the proportion of influenza B varied by country (data not shown).

Among genetically characterised influenza A(H1N1)pdm09 viruses, the majority (> 80%) belonged to the C.1.9 subclade of 5a.2a (Table 2). Most characterised influenza A(H3N2) viruses belonged to the J.2 subclade of 2a.3a.1. There was some genetic variation of influenza B viruses across studies, but all belonged to clade V1A.3a.2.

**Table 2:** Influenza viruses characterised by clade, amino acid substitutions and study site, five European studies, interim influenza season 2024/25 (n = 835)^a^

Characterised viruses	Clade	Subclade	DK-H/DK-PC^b^		EU-H		EU-PC		UK-PC	
			n	%	n	%	n	%	n	%
Influenza A(H1N1)pdm09			n = 564^c^		n = 212^c^		n = 712^c^		n = 2,444^c^	
A/Lisboa/188/2023	5a.2a	C.1.9	76	83	13	NC	160	90	195	91
A/Michigan/62/2023	5a.2a	C.1.8	0	0	0	0	0	0	0	0
A/Victoria/4897/2022**^d^**	5a.2a.1	D	16	17	0	0	17	10	20	9
**Total (n=497)^e^**			**92**	**100**	**13**	**NC**	**177**	**100**	**215**	**100**
Influenza A(H3N2)			n = 389^c^		n = 66^c^		n = 187^c^		n = 292^c^	
A/Thailand/8/2022**^d^**	2a.3a.1	J	0	0	0	0	0	0	0	0
A/Sydney/856/2023	2a.3a.1	J.1	0	0	0	0	0	0	0	0
A/Croatia/10136RV/2023	2a.3a.1	J.2	20	31	4	NC	34	NC	31	NC
A/West Virginia/51/2024	2a.3a.1	J.2.1	41	63	1	NC	1	NC	2	NC
A/Lisboa/216/2023	2a.3a.1	J.2.2	4	6	0	0	3	NC	2	NC
A/France/IDF-IPP29542/2023	2a.3a.1	J.4	0	0	0	0	0	0	0	0
A/Finland/402/2023	2a.3a	G.1.3.1	0	0	0	0	1	NC	1	NC
**Total (n=145)^e^**			**65**	**100**	**5**	**NC**	**39**	**NC**	**36**	**NC**
Influenza B/Victoria			n = 721^c^		n = 84^c^		n = 639^c^		n = 184^c^	
B/Netherlands/10335/2023	V1A.3a.2	C.2	0	0	0	0	0	0	0	0
B/Moldova/2030521/2023	V1A.3a.2	C.3	0	0	0	0	0	0	1	NC
B/Stockholm/3/2022	V1A.3a.2	C.5	1	NC	0	0	0	0	1	NC
B/Catalonia/2279261NS/2023	V1A.3a.2	C.5.1	7	NC	2	NC	61	50	13	NC
B/Switzerland/329/2024	V1A.3a.2	C.5.6	9	NC	1	NC	29	24	9	NC
B/Guangxi-Beiliu/2298/2023	V1A.3a.2	C.5.7	11	NC	1	NC	33	27	8	NC
**Total (n=187)^e^**			**28**	**NC**	**4**	**NC**	**123**	**100**	**32**	**NC**

DK-H: Denmark hospital study; DK-PC: Denmark primary care study; EU-H: European Union hospital multicentre VEBIS study; EU-PC: European Union primary care multicentre VEBIS study; NC: not calculated (percentages not shown where denominators < 60); UK-PC: United Kingdom multicentre primary care study; VEBIS: Vaccine Effectiveness, Burden and Impact Studies.

^a^ Genetic characterisation results not available from the England, Northern Ireland and Scotland hospital studies.

^b^ DK-H and DK-PC samples are combined.

^c^ n: total numbers of viruses (sub)typed.

^d^ Strain included in the 2024/25 northern hemisphere influenza vaccine.

^e^ Total: total number of (sub)typed viruses sequenced.

## Vaccine effectiveness overall and against influenza A

In the primary care setting, VE for any influenza among all ages ranged from 40% to 53%, with lowest age-stratified VE among adults ≥ 65 years (null in EU-PC, 38% in UK-PC). In the hospital setting, the all-age any influenza VE was 34–52%. We append the detailed results for VE against influenza A in Supplementary Figure S1.

## Vaccine effectiveness against influenza A(H1N1)pdm09

**Figure 3:**
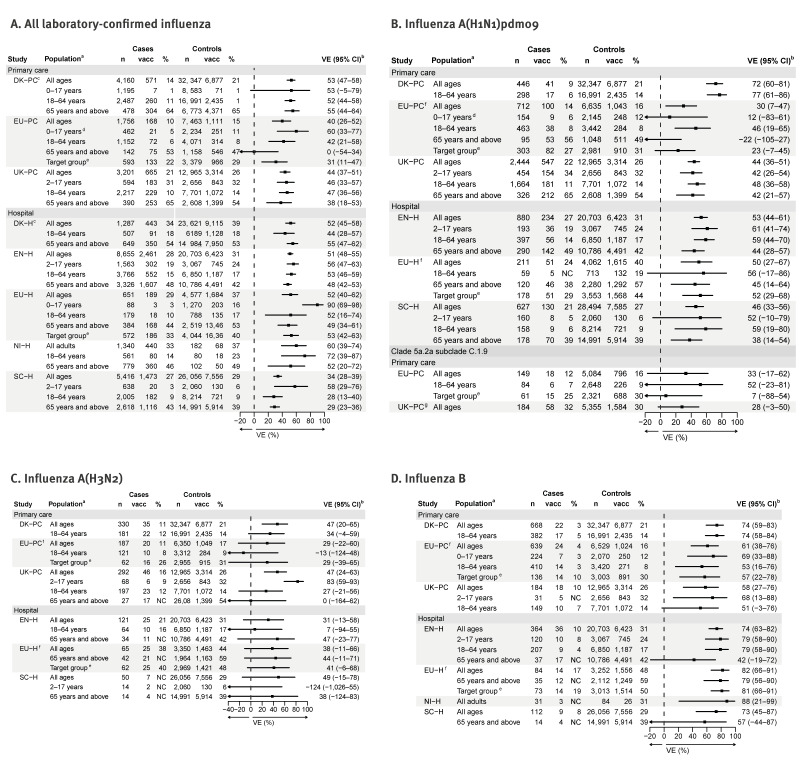
Interim vaccine effectiveness overall, against influenza A subtypes and influenza B, by age and target group for vaccination and by study, eight European studies, influenza season 2024/25

CI: confidence interval; DK-H: Denmark hospital study; DK-PC: Denmark primary care study; EN-H: England hospital study; EU: European Union; EU-H: EU hospital multicentre VEBIS study; EU-PC: EU primary care multicentre VEBIS study; NI-H: Northern Ireland hospital study; SC-H: Scottish hospital study; UK-PC: United Kingdom multicentre primary care study; VE: vaccine effectiveness; VEBIS: VE, Burden and Impact Studies.

^a^ Age-specific or target group-specific VE was not included for overall or (sub)type-specific VE in some study sites, where sample size did not allow estimation of VE. The definition of ‘all ages’ differs by study (Table 1), with patients ≥ 6 months-old in EU-PC, patients ≥ 2 years-old in UK-PC and EN-H, patients ≥ 18 years-old in NI-H and patients of all ages in DK-PC, DK-H, EU-H and SC-H.

^b^ For details of adjustment variables, see Table 1.

^c^ Estimates for DK-PC and DK-H are for any influenza A only, as numbers for influenza B were low.

^d^ For EU-PC: those aged 0–17 years are from ≥ 6 months to 17 years.

^e^ Groups targeted by seasonal influenza vaccination as defined locally in the studies and study sites.

^f^ For EU-PC: three study sites with < 10 influenza A(H1N1)pdm09 cases were excluded from A(H1N1)pdm09 VE analysis (3 cases); three study sites with < 10 influenza A(H3N2) cases were dropped from A(H3N2) VE analysis (8 cases); two study sites with < 10 influenza B cases were not included in B VE analysis (8 cases). For EU-H: one study site with < 10 influenza A(H1N1)pdm09 cases was excluded from A(H1N1)pdm09 VE analysis (3 cases); four study sites with < 10 influenza A(H3N2) cases were dropped from A(H3N2) VE analysis (6 cases); five study sites with < 10 influenza B cases were not included in B VE analysis (19 cases).

^g^ The A(H1N1)pdm09 5a.2a C.1.9-specific estimate for UK-PC includes England only.

The VE against influenza A(H1N1)pdm09 ranged between 30% and 72% among all ages in primary care settings. Age-specific results were similar among 18–64-year-olds in EU-PC and UK-PC at 46–48%, but higher in DK-PC at 77% (Figure 3). Age-specific VE was lower among children and adults ≥ 65 years in UK-PC (42% for both age groups) and even lower in EU-PC (12% and −22%), although confidence limits were wide. 

In the hospital setting across studies, VE against influenza A(H1N1)pdm09 ranged between 46% and 53% among all ages. The VE was lower in adults ≥ 65 years (38–45%) than among children aged 2–17 years (52–61%) (Figure 3).

Most sequenced samples of influenza A(H1N1)pdm09 belonged to the 5a.2a clade which, although genetically different to the vaccine strain, was protective for about one-third of those vaccinated among all ages (Figure 3). 

## Vaccine effectiveness against influenza A(H3N2)

The VE against influenza A(H3N2) ranged between 29% and 47% among all ages in primary care settings (Figure 3), while VE in adults ranged between −13% and 34%, with higher VE among children (83% in UK-PC).

In the hospital setting, VE against influenza A(H3N2) ranged between 31% and 49% among all ages. The VE in adults ranged from 7% to 47% and in SC-H (the only study with a VE estimate in children for this setting), VE among those aged 2–17 years was negative, noting that numbers were low. In particular, in SC-H, the proportion of influenza A cases subtyped (677/5,304; 13%) was very small (and only 50/677 (7%) of those subtyped were influenza A(H3N2)). There is a potential for some bias towards subtyping of more severe cases in this study as in some health boards in Scotland, patients admitted to intensive care or high-dependency wards are more likely to have their samples subtyped.

## Vaccine effectiveness against influenza B

The VE against influenza B was high across all studies, with all-age estimates ranging between 58% and 74% in primary care and 73–88% in hospital settings (Figure 3). Age-specific estimates were all ≥ 50%, except one (42%, among those ≥ 65 years in EN-H). 

## Discussion

The influenza epidemic is ongoing in Europe [11,12]. Results from eight European influenza studies in the early phase of the winter 2024/25 influenza season indicated that influenza vaccination prevented from one-third to more than three-quarters of influenza infections medically attended in the primary care or hospital settings among the vaccinated, although protection varied by age group and study. Canadian interim 2024/25 VE in the primary care setting was in between our European study estimates at 50–57% [13].

This season, C.1.9, harbouring the K169Q amino acid substitution, is the main circulating A(H1N1)pdm09 5a.2a subclade. The influenza A(H1N1)pdm09 clade 5a.2a also dominated in Europe last season [14], and the vaccine, unchanged since 2023/24, provided protection during the 2023/24 interim A(H1N1)pdm09 VE season similar to or higher than we present for interim 2024/25 season VE [6,15]. The 2023/24 clade-specific VE point estimate for primary care settings was higher than in the current season (52% vs 28–33% among all ages) [15], indicating that virological change within the 5a.2a clade may be causing immune escape. Some VE estimates by age group were low, although antigenic studies with ferret sera indicate that circulating 5a.2a viruses are generally well recognised by sera raised against the clade 5a.2a.1 vaccine virus [14]. Repeat vaccination may play a role in lower VE, as previously hypothesised in some studies [16]; however, small sample size affecting the point estimates may be more likely.

In general, VE against influenza A(H3N2) in the primary care setting was lower than the 54% observed in Canada in this 2024/25 season [13], but due to low circulation of influenza A(H3N2) in Europe to date, all VE estimates had wide confidence intervals. The VE estimates later in the season with more cases will help confirm the VE against circulating A(H3N2) viruses which, although clade-matched to the clade 2a.3a.1 vaccine virus, harbour some genetic variation. Antigenic studies indicate reduced reactivity of ferret antibodies raised against the vaccine virus with the circulating viruses [14].

Circulating influenza B strains were clade-matched to the clade V1A.3a.2 vaccine virus, which had remained unchanged since the 2022/23 northern hemisphere season. The VE was similarly high in the 2022/23 and 2023/24 seasons [8,9,15,17]. 

These influenza VE results should contribute to the supporting evidence for the WHO composition meeting for the northern hemisphere influenza vaccine strain selection on 24–27 February 2025. 

## Conclusion

Influenza vaccination should continue to be promoted in target groups, where feasible, as vaccination against influenza A, the main circulating influenza type, protected from one-third to over one-half of vaccinated individuals. Given that there were signals of lower VE by subtype, and particularly among older adults in some studies, in this time of heightened influenza activity other infection prevention measures should also be strengthened. End-of-season influenza VE with greater sample size, combined with more information on genetic variation of viruses, may help clarify observed differences in age- and study-specific VE. 

## Supplementary Data

336000 Supplement
