# Erratum for Euro Surveill. 2025;30(7)

**DOI:** 10.2807/1560-7917.ES.2025.30.11.240320c

**Published:** 2025-03-20

**Authors:** Eurosurveillance editorial team

**Affiliations:** 1 European Centre for Disease Prevention and Control (ECDC), Stockholm, Sweden

In the article ‘Interim 2024/25 influenza vaccine effectiveness: eight European studies, September 2024 to January 2025’ by Rose et al, published on 20 February 2025, the following changes were made on 20 March 2025 at the request of the authors: The ID number of the Croatian ethical approval under EU-PC was corrected. The following names were added to the Acknowledgements: Maja Ilić, Ivana Ferenčak, Katica Čusek Adamić, Mirjana Lana Kosanović Ličina, Ivana Mihin Huskić, Diana Nonković, Morana Tomljenović. The spelling of the name of one European IVE group member under Collaborators was corrected.

