# Supplementary material for: Interim 2024/25 influenza vaccine effectiveness: eight European studies, September 2024 to January 2025
Source: Euro Surveill. 2025 Feb 20;30(7):2500102. doi: 10.2807/1560-7917.ES.2025.30.7.2500102 (PMC11843620; doi:10.2807/1560-7917.ES.2025.30.7.2500102)
Supplement: Supplement [file 25-00102_ROSE_Supplement.pdf]

Supplementary material

This supplementary material is hosted by *Eurosurveillance* as supporting information alongside the article "Interim 2024/25 influenza vaccine effectiveness: eight European studies, September 2024 to January 2025", on behalf of the authors, who remain responsible for the accuracy and appropriateness of the content. The same standards for ethics, copyright, attributions and permissions as for the article apply. Supplements are not edited by *Eurosurveillance* and the journal is not responsible for the maintenance of any links or email addresses provided therein.

Supplementary Figure S1. Interim adjusted vaccine effectiveness (VE) against all laboratory-confirmed influenza A, by age and target group for vaccination and by study, eight European studies, influenza season 2024/25

Influenza A

Study	Population[a]	Cases N	Cases vacc	Cases %	Controls N	Controls vacc	Controls %	VE (95% CI)[b]
PC								
DK-PC	All ages	4160	571	14	32347	6877	21	53 (47–58)
	0–17 years	1195	7	1	8583	71	1	53 (−5–79)
	18–64 years	2487	260	11	16991	2435	1	52 (44–58)
	65 years and above	478	304	64	6773	4371	65	55 (44–64)
EU-PC	All ages	1114	146	13	7450	1111	15	32 (14–46)
	0–17 years[d]	238	15	6	2227	251	11	35 (−18–66)
	18–64 years	739	59	8	4066	314	8	38 (13–56)
	65 years and above	137	72	53	1157	546	47	1 (−53–35)
	Target group[e]	457	120	26	3371	966	29	24 (0–43)
UK-PC	All ages	3023	649	22	12965	3314	26	43 (35–49)
	2–17 years	565	179	32	2656	843	32	44 (30–55)
	18–64 years	2071	219	11	7701	1072	14	46 (34–55)
	65 years and above	387	251	65	2608	1399	54	37 (17–53)
Hospital								
DK-H	All ages	1287	443	34	23621	9115	39	52 (45–58)
	18–64 years	507	91	18	6189	1128	18	44 (28–57)
	65 years and above	649	350	54	14984	7950	53	55 (47–62)
EN-H	All ages	8304	2427	29	20703	6423	31	51 (47–54)
	2–17 years	1447	293	20	3067	745	24	55 (46–63)
	18–64 years	3565	543	15	6850	1187	17	52 (45–58)
	65 years and above	3292	1591	48	10786	4491	42	48 (43–53)
EU-H	All ages	512	159	31	4408	1648	37	47 (32–58)
	0–17 years	54	1	NC	1233	200	16	92 (60–100)
	18–64 years	134	15	11	758	134	18	41 (−10–70)
	65 years and above	324	143	44	2417	1314	54	45 (28–59)
	Target group[e]	455	157	35	3897	1601	41	48 (34–60)
NI-H	All adults	1309	437	33	98	42	43	56 (31–72)
	18–64 years	536	79	15	48	14	29	71 (37–87)
	65 years and above	773	358	46	50	28	56	52 (19–72)
SC-H	All ages	5304	1464	28	26056	7556	29	33 (28–38)
	2–17 years	601	19	3	2060	130	6	59 (29–76)
	18–64 years	1947	181	9	8214	721	9	27 (12–39)
	65 years and above	2604	1112	43	14991	5914	39	29 (22–36)

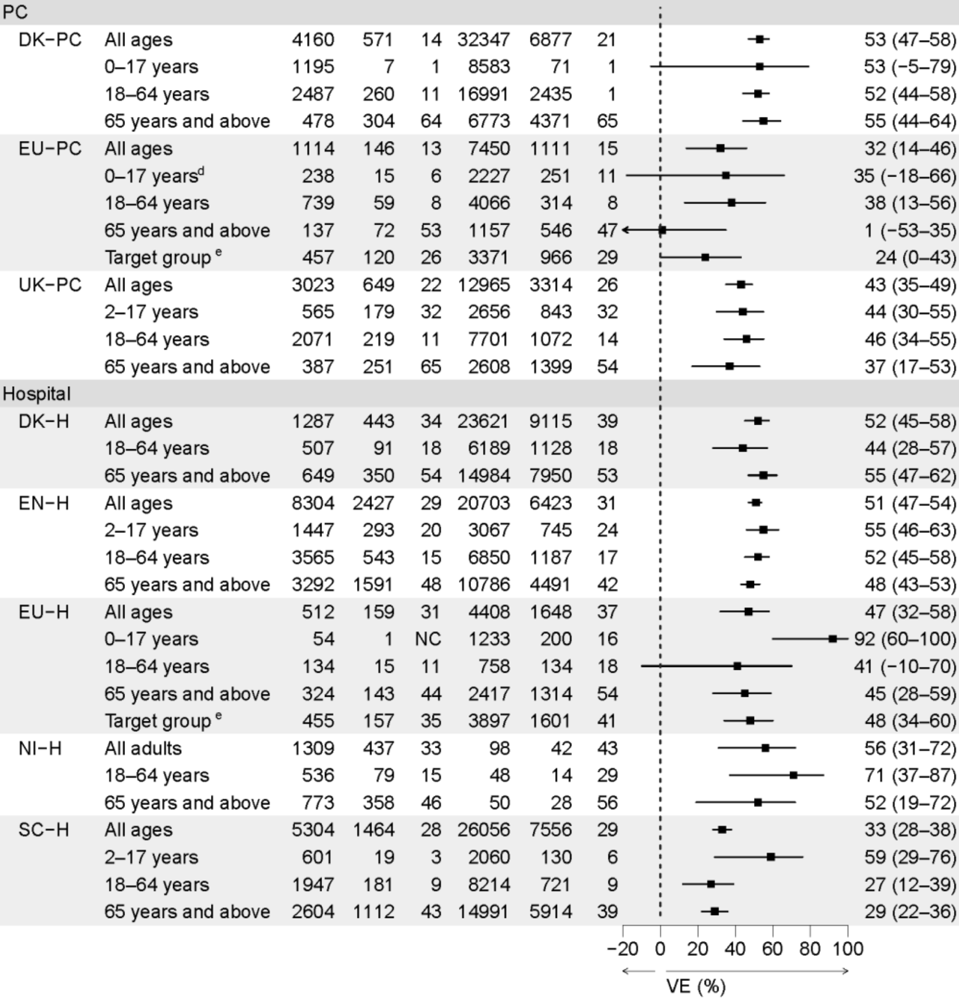

CI: confidence interval; DK-H: Denmark hospital study; DK-PC: Denmark primary care study; EN-H: England hospital study; EU: European Union; EU-H: EU hospital multicentre VEBIS study; EU-PC: EU primary care multicentre VEBIS study; PC: primary care; NI-H: Northern Ireland hospital study; SC-H: Scottish hospital study; UK-PC: United Kingdom multicentre primary care study; VE: vaccine effectiveness; VEBIS: VE, Burden and Impact Studies.

[a] Age-specific or target group-specific VE was not included in some study sites, where sample size did not allow estimation of VE. The definition of “all ages” differs by study (Table 1), with patients ≥ 6 months old in EU-PC, patients ≥ 2 years old in UK-PC and EN-H, patients ≥ 18 years old in NI-H and patients of all ages in DK-PC, DK-H, EU-H and SC-H.

[b] For details of adjustment variables, see Table 1.

[c] For EU-PC: those aged 0–17 years in the table are from ≥6 months to 17 years.

[d] Groups targeted by seasonal influenza vaccination as defined locally in the studies and study sites.
